# Macrophages: The Good, the Bad, and the Gluttony

**DOI:** 10.3389/fimmu.2021.708186

**Published:** 2021-08-12

**Authors:** Ewan A. Ross, Andrew Devitt, Jill R. Johnson

**Affiliations:** School of Biosciences, College of Health and Life Sciences, Aston University, Birmingham, United Kingdom

**Keywords:** macrophage, inflammation, arthritis (including rheumatoid arthritis), asthma, apoptosis

## Abstract

Macrophages are dynamic cells that play critical roles in the induction and resolution of sterile inflammation. In this review, we will compile and interpret recent findings on the plasticity of macrophages and how these cells contribute to the development of non-infectious inflammatory diseases, with a particular focus on allergic and autoimmune disorders. The critical roles of macrophages in the resolution of inflammation will then be examined, emphasizing the ability of macrophages to clear apoptotic immune cells. Rheumatoid arthritis (RA) is a chronic autoimmune-driven spectrum of diseases where persistent inflammation results in synovial hyperplasia and excessive immune cell accumulation, leading to remodeling and reduced function in affected joints. Macrophages are central to the pathophysiology of RA, driving episodic cycles of chronic inflammation and tissue destruction. RA patients have increased numbers of active M1 polarized pro-inflammatory macrophages and few or inactive M2 type cells. This imbalance in macrophage homeostasis is a main contributor to pro-inflammatory mediators in RA, resulting in continual activation of immune and stromal populations and accelerated tissue remodeling. Modulation of macrophage phenotype and function remains a key therapeutic goal for the treatment of this disease. Intriguingly, therapeutic intervention with glucocorticoids or other DMARDs promotes the re-polarization of M1 macrophages to an anti-inflammatory M2 phenotype; this reprogramming is dependent on metabolic changes to promote phenotypic switching. Allergic asthma is associated with Th2-polarised airway inflammation, structural remodeling of the large airways, and airway hyperresponsiveness. Macrophage polarization has a profound impact on asthma pathogenesis, as the response to allergen exposure is regulated by an intricate interplay between local immune factors including cytokines, chemokines and danger signals from neighboring cells. In the Th2-polarized environment characteristic of allergic asthma, high levels of IL-4 produced by locally infiltrating innate lymphoid cells and helper T cells promote the acquisition of an alternatively activated M2a phenotype in macrophages, with myriad effects on the local immune response and airway structure. Targeting regulators of macrophage plasticity is currently being pursued in the treatment of allergic asthma and other allergic diseases. Macrophages promote the re-balancing of pro-inflammatory responses towards pro-resolution responses and are thus central to the success of an inflammatory response. It has long been established that apoptosis supports monocyte and macrophage recruitment to sites of inflammation, facilitating subsequent corpse clearance. This drives resolution responses and mediates a phenotypic switch in the polarity of macrophages. However, the role of apoptotic cell-derived extracellular vesicles (ACdEV) in the recruitment and control of macrophage phenotype has received remarkably little attention. ACdEV are powerful mediators of intercellular communication, carrying a wealth of lipid and protein mediators that may modulate macrophage phenotype, including a cargo of active immune-modulating enzymes. The impact of such interactions may result in repair or disease in different contexts. In this review, we will discuss the origin, characterization, and activity of macrophages in sterile inflammatory diseases and the underlying mechanisms of macrophage polarization *via* ACdEV and apoptotic cell clearance, in order to provide new insights into therapeutic strategies that could exploit the capabilities of these agile and responsive cells.

## Macrophages in Sterile Inflammation

Macrophages were first described by Élie Metchnikoff in 1883, following microscopic observations of mobile cells responding to injury in a starfish larva induced by the insertion of a thorn (1). These cells were subsequently classified according to their size, designated as macrophages (“big eaters”) and microphages (“small eaters”, now known as neutrophils) (2). Early studies postulated that macrophages are tissue-derived cells closely associated with the vascular endothelium, but experiments in the late 1960s demonstrated that at least some macrophages differentiate from monocytes in the blood circulation (3). Eventually, these theories coalesced into the notion of a dual origin of tissue macrophages, in that tissues are seeded during development with primitive macrophages from the fetal liver and yolk sac, representing the tissue-resident macrophage population, while in adulthood, macrophages also develop from bone marrow-resident hematopoietic stem cells, with monocytes in the blood as an intermediate cell type (4).

Macrophages exhibit a considerable degree of plasticity depending on signals from the extracellular environment, defined by the M1 *vs.* M2 continuum of macrophage polarization. Macrophages on the M1 end of the spectrum are considered a pro-inflammatory phenotype, with robust phagocytic and cytotoxic capacity; these cells are defined by their expression of major histocompatibility complex (MHC) Class II, cluster of differentiation (CD)14, CD80/CD86, and CD38, as well as inducible nitric oxide synthase (iNOS). M1 macrophages robustly express pro-inflammatory cytokines (e.g., IL-6, IL-12, IL-1β, and TNF-α) and chemokines (e.g., CCL2, CCL5), reflective of their ability to recruit other immune cells (T cells, B cells) to the site of infection and maintain their activation (4). Conversely, M2 macrophages function in the resolution of inflammation and tissue repair pathways, and express the cell surface markers CD36, CD206, and CD163 (5). Compared to M1 macrophages, M2 macrophages are more functionally diverse, with several subtypes (M2a, M2b, M2c, M2d) expressing different combinations of cytokines, chemokines, and growth factors (4, 6). M2a macrophages, closely associated with Th2 polarized allergic inflammation in the lung, are induced by IL-4 and/or IL-13 and express high levels of IL-10, TGF-β, and inflammatory chemokines such as CCL17, CCL18, CCL22, and CCL24. Conversely, M2b macrophages are promoted by immune complexes and have been shown to play important roles in Th2 immune responses *via* their expression of TNF-α, IL-1β, IL-6, IL-10, and CCL1. Subsequently, a microenvironment rich in IL-10 and prostaglandin E2 leads to the induction of M2c macrophages, which continue to express IL-10 as well as TGF-β, and thereby are key regulators of the resolution of inflammation and tissue repair. Finally, M2d macrophage arise in response to TLR and adenosine A2A receptor ligands, as well as IL-6, and have been shown to participate in angiogenesis *via* the expression of vascular endothelial growth factor (VEGF) and IL-10 (7–9). However, it is important to note that the polarization state of macrophages is in fact a continuum, as these cells are able to adopt intermediate phenotypes, with heterogeneous subpopulations taking on a variety of physiological roles. A major challenge in the field, however, is the mechanism by which, especially in humans, macrophage phenotypes are defined. The simple approach to define M1 and M2 as poles of a continuum presents challenges for the comparison of different studies, particularly in studies assessing macrophages generated and/or matured *in vitro*; in fact, it has been suggested by some authors that researchers describe stimulation parameters as an aspect of macrophage nomenclature, i.e. M(IL-4) rather than M2, in an effort to provide additional clarity with regard to experimental conditions and to allow more transparent comparisons between studies (10). It is also understood that so-called “markers” of M1 and M2 (e.g. CD163, TNF-α, CD209 and TGF-β) can be co-expressed by individual cells, highlighting the complexity of polarization states (11).

**Figure 1:**
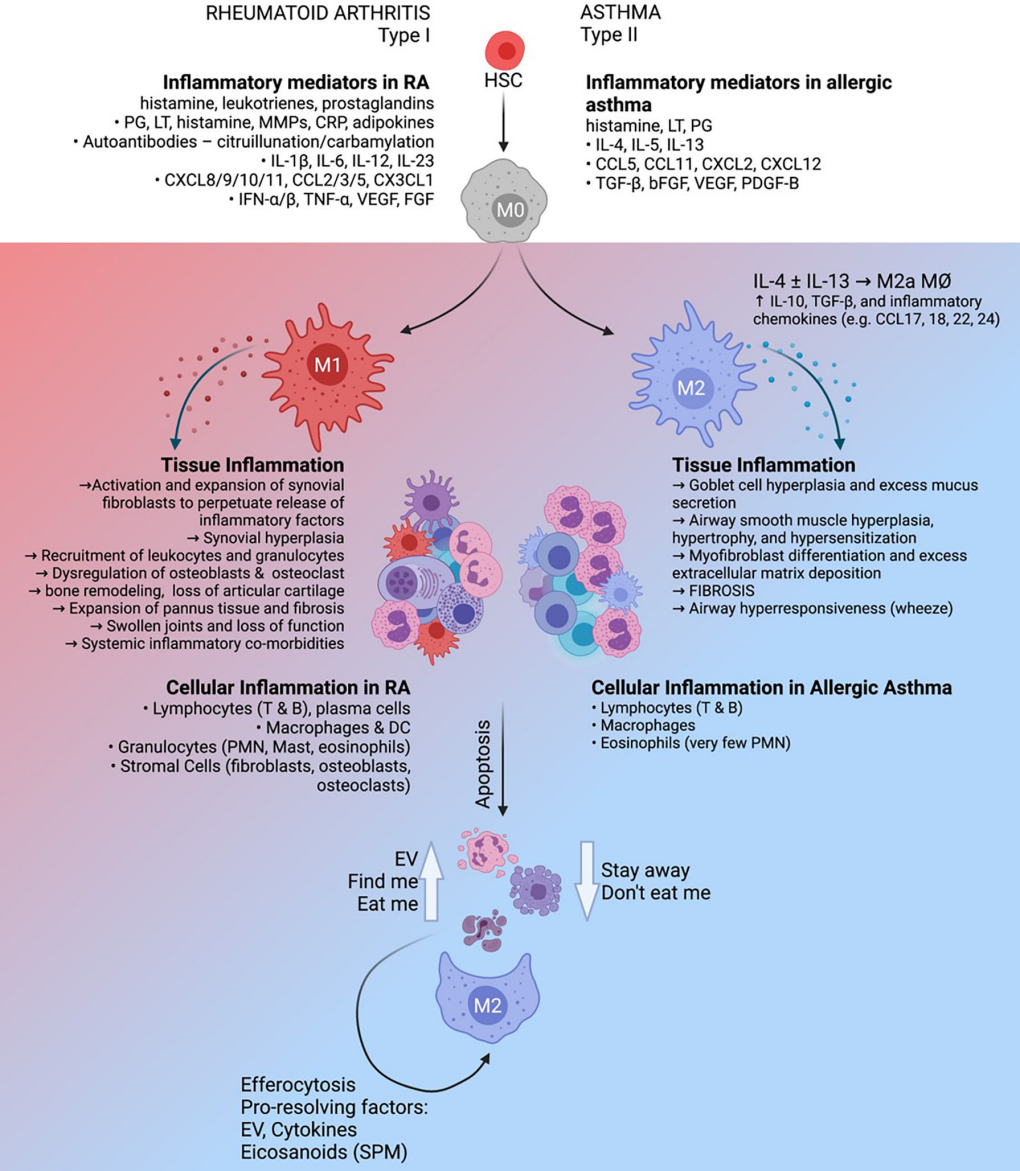
The roles and regulatory capacity of macrophages in chronic sterile inflammation. Rheumatoid arthritis (left) is characterized as a Th1-polarized immune pathology in the articular joints which can be driven by autoimmune factors such as citrullinated/carbamylated autoantibodies, whereas allergic asthma (right) is a Th2-polarized inflammatory response to inhaled allergen in the lung. These distinct inflammatory environments have profound effects on the activation and function of tissue-resident macrophages, which in turn play key roles in maintenance of the diseased state. Clearance of inflammatory cells from the tissue by phagocytic macrophages (efferocytosis), supported by extracellular vesicles produced by apoptotic cells, has the potential to support the resolution of inflammation.

It is currently well-understood that macrophages are dynamic cells with critical roles in the induction and resolution of sterile inflammation (**Figure 1**). In this review, we will compile and interpret recent findings on the plasticity of macrophages in two non-infectious, chronic inflammatory diseases with contrasting immunological profiles: rheumatoid arthritis as a representative Th1-associated disease and allergic asthma as a classic Th2-skewed pathology. Furthermore, the critical roles of macrophages in the resolution of tissue inflammation will also be examined, emphasizing the ability of macrophages to clear apoptotic immune cells and contribute to the resolution of sterile inflammation.

## The Role of Macrophages in the Induction and Maintenance of Inflammation: Rheumatoid Arthritis

### The Immune Response in Rheumatoid Arthritis

Rheumatoid arthritis (RA) is a chronic systemic autoimmune condition which primarily affects the articular joints (12). This chronic inflammatory disease is one of the most common autoimmune conditions, affecting 0.5-1% of the world’s population, with a higher incidence in women compared to men that increases in the elderly population (13). It is characterized by swollen and painful joints at presentation, which are a result of thickening of the synovial lining layer, loss of articular cartilage and bone remodeling leading to lack of function or deformation. Due to the high levels of systemic inflammation, comorbidities often perturb the function of other organs such as blood vessels, kidneys, heart and lungs. To date, no one specific trigger has been identified to induce RA; however, multiple risk factors have been identified, including genetic predisposition (over 100 associated polymorphisms have been identified contributing to approximately 30-60% of the overall risk), epigenetic changes and environmental factors (12). RA occurs through the loss of tolerance to self-antigens and the generation of autoantibodies to post-translationally modified proteins *via* processes such as citrullination or carbamylation (13). Critically, these autoantibodies can be detected in patients prior to the development of joint inflammation (14). Indeed, there is no detectable infiltration or inflammation of the synovium in seropositive patients with anti-citrullinated antibodies who subsequently go on to develop RA at a later stage (15).

How the presence of these autoantibodies ultimately leads to symptomatic disease and presentation of joint swelling is not well-understood [recently reviewed in (16)]. The formation of immune complexes containing these autoantibodies is an important feature of the etiology of RA and drive many of the pathogenic features including complement activation and direct stimulation of immune cells (17–19). However, the disease process shifts to the joint environment where the activation of cells in the synovial lining layer are fundamental to establishing the inflammatory environment. Firstly, synovial fibroblasts are one of the main sources of IL-6 and MMPs, which degrade cartilage. Secondly, synovial inflammatory macrophages are the main source of pathogenic cytokines, including TNF-α (12). Cell-cell interactions between macrophages and fibroblasts have been demonstrated to be critical in amplifying and driving disease progression (20, 21). Disease onset leads to the recruitment of monocytes from the circulation where they differentiate locally into pro-inflammatory macrophages; these cells are the main source of chronicity factors such as TNF-α, IL-1, IL-6 and GM-CSF (22). Over time, thickening of the synovium occurs through the expansion of fibroblast and macrophage populations, which alters the synovial environment. Excessive production of soluble factors that recruit lymphocytes and granulocytes to the inflamed joint, as well as inducing their proliferation and retention, but blocking apoptosis, leads to a self-perpetuating state of inflammation (23). The success of therapies targeted to myeloid-derived soluble factors and the use of models of disease have revealed that this loss of homeostasis and the dysregulated synovial inflammatory environment is maintained through the recruitment of pro-inflammatory macrophages to the synovium (22, 24).

### Macrophages In Healthy Joints

In healthy joints, the synovial membrane is a multifunctional connective tissue structure found on the inner surface of the joint capsule in contact with the lubricating synovial fluid. This synovial layer is normally very thin (approximately 2 mm) and can be clinically assessed using ultrasound measurements (24). It is formed by two distinct cell layers whose main roles are to maintain homeostasis within the joint, secrete lubricating fluids as well as controlling access to the joint space for peripheral blood cells. The synovial lining layer is composed of two main cell types; synovial lining fibroblasts (SFibro) and resident synovial lining macrophages (SMacs) (25). The second supporting layer consists of sub-lining fibroblasts, connective tissue, blood vessels, lymphatic drainage and nerves to support the functions of the lining layer. Monocytes are almost never detected in healthy synovial fluid suggesting the SMacs are not replenished by emigration of their precursors (26). This is supported by observations from murine studies, where most organs contain a prenatal macrophage population which are required to maintain tissue homeostasis (27, 28). Indeed, populations of resident macrophages have been described in the synovium which are derived through seeding by similar prenatal populations during development (29, 30). In mice, SMacs can proliferate *in situ* and importantly differ from infiltrating monocyte derived macrophages (MDM) from the circulation. Tissue resident cells have a Krüppel-like factor 2 (KLF2)/KLF4 transcription program which both mediates apoptotic cell uptake and inhibits pro-inflammatory TLR signaling (31). However these resident macrophages are only constitute a proportion of the total macrophage population present in the joint and as such have been difficult to distinguish from monocyte derived macrophages until recently. For example, a recent elegant study from Culemann and colleagues (30) revealed that these cells can be marked by the surface expression of fractalkine receptor (CX3CR1^+^) and co-stained for macrophage markers including CD68 and F4/80. Importantly the authors demonstrated these cells expanded during auto-antibody induced inflammatory arthritis and were seeded during development using reporter models. This CX3CR1^+^ population maintains tight junction formation in the healthy synovium, preventing cell ingress (30). Redistribution of these cells at the onset of inflammation results in weakening of this protective barrier, allowing the infiltration of pro-inflammatory MDM from the circulation into the synovium (32).

Distinguishing between tissue resident and monocyte derived macrophages is difficult and has been investigated in other tissue sites, informing on markers or physiology that may be informative to help define these rare synovial cells. Fate mapping to determine the origin of these cells during development has allowed the definition of these cells in multiple tissues such as the gut, dermis and heart (28). For example, expression of GATA-6 identifies tissue resident macrophages which self-renew in models of peritonitis; co-expression of combinations of CD11b, F4/80 and CD64 markers identify subsets of these resident cells from monocyte derived macrophages (33). These tissue resident peritoneal cells constantly assess their environment, monitoring for tissue damage and promoting a pro-resolution state to maintain homeostasis (34). Whilst these peritoneal cells have some similar physiological functions to those described in resident synovial macrophages, given the high degree of tissue-specific transcriptional, physiological and epigenetic effects observed on macrophages (28, 35), more detailed work on specific synovial specific populations is still required to understand their therapeutic potential. Currently, macrophage phenotypic markers including TREM2, CD48, LYVE1 and CLEC10A have been identified to begin to stratify distinct tissue resident subsets in the synovium using combinations of lineage tracing and transcriptomics (36–38).

SMacs are a heterogenous population consisting of several distinct subsets; recent findings reveal that changes in the population dynamics within the SMac population can be observed at the onset of inflammation [recently reviewed in (39)]. In human healthy joints, SMacs express the scavenger receptor marker CD163 and are also strongly phagocytic (40, 41). SMacs engulf and remove apoptotic cells, and this has been shown to reduce inflammation by reducing recruitment of neutrophils to the synovium (42). Indeed, a specific polymorphism in the scavenger receptor VSIG4 has recently been described to be a strong risk factor correlate for severe disease in RA (43). They also express the markers MHCII and osteoprotegerin, but lack pro-inflammatory cytokines (TNF-α, IL-1β) or RANKL, required to induce bone resorption (40, 41). Recently resident SMacs have also been shown to express myeloid-epithelial-reproductive tyrosine kinase (MerTK), CD206 and triggering receptor expressed on myeloid cells (TREM2) (36). These SMacs are postulated to play a similar role to murine CX3CR1^+^ SMacs as they express several tight junction-related genes. Thus, although human SMacs are still not as well understood as murine counterparts, they appear to have a similar protective phenotype required to maintain normal homeostasis of the joint tissue.

### Macrophages in Rheumatoid Arthritis

Macrophages are one of the principal drivers of both inflammation and chronicity in the joint of RA patients where they secrete many of the factors closely associated with this disease; pro-inflammatory cytokines (TNF-α, IL-1 and IL-6), chemoattractants (CCL2 and IL-8) as well as tissue remodeling enzymes (MMP-3 and MMP-12) (44). In RA patients, a hypertrophic synovial lining layer develops due to fibroblast proliferation, increased vasculature and infiltration of MDM from the circulation. The degree of synovitis is directly related to recruitment of monocytes (44), causing an increase in the total synovial macrophage population (45). This accumulation correlates to disease activity and is used as part of a biomarker assessment to assess the efficacy of therapeutic interventions (46, 47). These observations have been experimentally investigated using various mouse models of RA (48). In humans, clinical studies of radiolabeled CD14^+^ monocytes revealed their active migration into an inflamed joint, where they polarize to an inflammatory MDM within the inflamed synovium (49, 50). Synovitis can be defined by histological analysis where distinct disease activity subtypes are identified depending on the cellular infiltrate present [reviewed in (39)].

The hypertrophic synovium reduces oxygen tension within the joint to hypoxic levels, which can fall below 1% (51). This environment alters macrophage respiration through upregulation of HIF-1α, reducing oxidative phosphorylation and promoting anaerobic glycolysis which supports their pro-inflammatory activation *in situ* (52, 53). This environmental effect has been demonstrated in a myeloid specific HIF-1α deletion transgenic that results in reduced inflammation and joint swelling in a murine model of arthritis (54). To date, two distinct populations of blood-derived pro-inflammatory monocytes have been described to traffic into the inflamed synovium in models of RA, driving expansion of macrophage numbers. Classical Ly6C^+^ monocytes drive inflammation in adjuvant and antigen-induced arthritis whilst recruitment of non-classical Ly6C^-^ cells occurs in sterile models of autoantibody-induced arthritis (29, 55). Ly6C^-^ cells will traffic to the site of inflammation, and during the effector phase, differentiate into an M1 type pro-inflammatory classically activated macrophage which drives pathology without altering the number of SMacs. Interestingly the same population of activated macrophages can subsequently change their polarization to a non-classical M2 anti-inflammatory phenotype which are necessary for the resolution of the inflammation (29). This plasticity in response suggests that conversion of pro-inflammatory macrophages to a pro-resolution phenotype may be a viable therapeutic strategy in human disease.

### Macrophage Subsets in Rheumatoid Arthritis

Early histological studies of inflamed patient tissue identified distinct subsets of macrophages in the synovium depending on labeling by combinations of individual antibody clones (56). The frequency of these distinct subsets was found to change depending on levels of disease activity in the tissue examined. In active disease, pro-inflammatory MDMs constitute the majority of synovial macrophages (49), and when isolated from inflamed tissues of RA patients with active disease release proinflammatory mediators (such as TNF, IL-1, IL-6, CXCL8 and CCL2) (57–59) and were able to stimulate autologous T cells (60). The pro-inflammatory nature of RA MDM is now well-established, with secretion of CXCL4 and CXCL7 to attract neutrophils and monocytes to the inflamed joint (61) and release of cytokines such as TNF-α, IL-1, IL-6, GM-CSF and TGF-β are well-documented (62–64). Infiltrating MDM in the synovium can be identified with an antibody against an epitope on the alarmins S100A8/9 (65), and are susceptible to anti-TNF therapy, leading to their rapid removal (49, 66). In contrast, SMacs are not affected by anti-TNF treatment (66), demonstrating that specific targeting of MDMs may be possible as a treatment strategy.

As technology has progressed, the complexity of this heterogenous mixture of macrophages in the synovium during disease is being revealed. Using refined ultrasound guided tissue biopsy techniques of synovium in combination with single-cell transcriptomics approaches have provided more consistent tissue samples and given further insight into the heterogeneity of this population (37, 38, 67). Recently, distinct clusters of SMacs or MDMs with diverse homeostatic, inflammatory, resolving or regulatory functions have been identified (36). Importantly, protective or pro-repair macrophages (MerTK^+^/CD206^+^) could be distinguished from those with pro-inflammatory gene signatures (MerTK^-^/CD206^-^). This study has further suggested that these two distinct functional subsets can be further stratified into a total of nine distinct populations based on gene expression patterns. Intriguingly, the frequency of these populations changes depending on disease activity. For example, a subset of CD163^+^/MerTK^+^/CD206^+^ macrophages were greatly increased in RA patients in remission compared to those with active disease. Understanding how these individual subsets react and change between disease and remissive states will be crucial for our understanding of which subsets to target and influence in order to drive an anti-inflammatory, pro-resolving therapy.

In RA, macrophages in the joint are constantly influenced by the inflammatory environment to further sustain levels of pro-inflammatory and cell recruitment factors, whilst also acting to promote bone erosion and vascularization. As well as cytokines and chemokines, synovial tissue and fluid macrophages have the ability to respond to PAMPs and DAMPs. Activation of sMACs or their physiological responses can be modulated through ligation of activatory or inhibitory receptors such as Fc or TLRs to further promote the pro-inflammatory phenotype and sustain the chronic inflammatory environment (68, 69). Given the sterile nature of the chronic phase of the disease, DAMPs derived from endogenous or altered self-proteins have been described to be associated with inflammation in RA (70). DAMPs such as intracellular molecules, nucleic acids released from damaged or necrotic cells, matrix fragments or oxidized lipoproteins are found at increased levels at sites of inflammation (71). Macrophages have a variety of pattern recognition receptors (PRRs) on their surface which, when ligated with DAMP molecules, promote phagocytosis, the release of inflammatory mediators as well as antigen presentation and thus can both drive and sustain an inflammatory response (72). RA patients have detectable DAMPs including heat shock proteins, S100 proteins, HMBG1 and citrullinated histones in synovial fluid (73–75). In the RA synovium itself, DAMPs such as heat shock proteins, fibronectin fragments and tenascin-C have been identified, further potentially directly fueling macrophage pro-inflammatory activity (76, 77). For example, tenascin-C induces the secretion of IL-1β and TNFα in sMacs ex vivo after ligating TLR4 (78, 79). Directly injecting tenascin-C into a healthy joint drives inflammation and, conversely, animals deficient in tenascin-C are protected from erosive arthritis (80). RA sMacs have increased expression of TLR2 and TLR4 on their cell surface and when stimulated with DAMP ligands ex vivo, and secrete higher levels of pro-inflammatory factors (81, 82). This correlates with a recent observation that synovial macrophages from established RA have a transcriptome profile similar to that observed in cells activated by pathogens (83). Sensitivity to these ligands can be further enhanced through exposure to oxidized oxysterols which are enriched in RA synovial fluid, resulting in increased secretion of pro-inflammatory factors (84). In addition, activation of TLR4 can promote the survival and proliferation of RA macrophages through activation of the nuclear factor of activated T cells 5 (NFAT5) signaling pathway (85). Therefore, DAMPs generated during the inflammatory response through tissue damage or cellular stress in RA provide a source of factors that can both sustain and enhance sMac pro-inflammatory behavior.

Macrophages in active RA joints also display dysregulated expression of inhibitory and stimulatory Fc receptors, leading to increased secretion of TNF-α ex vivo (86, 87). Fc receptors will bind to autoantibodies to form immune complexes present in the peripheral blood serum, synovial fluid and synovial tissue of seropositive RA patients (19, 88, 89). Immune complexes comprising autoimmune antibodies including anti-citrullinated protein antibodies (ACPA) or rheumatoid factor (RF) have been demonstrated to not only induce complement activation, leading to increased cell recruitment to the joint (17, 18), but can also directly activate macrophages to secrete pro-inflammatory factors. Cross-linking of the FcγRIIa receptor with RA immune complexes results in robust TNFα secretion and an amplification of the pro-inflammatory profile of macrophages (90, 91). Priming of macrophages with macrophage colony stimulating factor (M-CSF) enhances this effect and the synovial fluid of RA patients contains large amounts of this cytokine, further increasing the pro-inflammatory potential of these cells (92, 93). Co-stimulation of macrophages through TLR and Fc receptors with IgG-immune complexes containing citrullinated matrix proteins can further enhance TNF-α secretion (90, 91, 94). M2 type RA macrophages, when co-stimulated *via* Fc and TLR with these immune complexes, lose their ability to reduce inflammation and instead secrete pro-inflammatory factors such as IL-6, TNFα and IL-1β (69, 95). This co-stimulatory effect demonstrates the ability of the dysregulated environment to alter or amplify the sMac phenotype to further drive this chronic inflammatory state. Given the dysregulated expression of these receptors, targeting sMacs *via* Fc receptor expression has been proposed as a potential therapeutic methodology to deplete myeloid populations and thereby reduce inflammation in RA (96).

Macrophages in the synovium predominantly secrete the chemokines CXCL4 and CXCL7 in early stage of RA to recruit neutrophils and monocytes (61), but will produce pro-inflammatory mediators TNF-α (63), IL-1β (62), IL-6 (64) and alarmins S100A8/9 (66) throughout active disease. Continued secretion of these factors promotes the constant recruitment and activation of leukocytes into the joint (97), and is maintained through the transcription factors NF-κB, IRF5, STAT1/5 (98, 99) as well as modifiers of gene expression such as microRNA-155 (100). Pro-inflammatory macrophages also secrete angiogenic factors such as VEGF or TGF-α/β to increase new blood vessel formation (101), as well as influencing fibroblasts to secrete RANKL, leading to bone erosion (99). In this context, macrophages may also be able to directly differentiate into mature osteoclasts given the correct environmental cues such as CCL25/CCR9 to further increase bone erosion (102, 103). This concept is supported by the identification of an osteoclastogenic macrophage subset in the synovial tissue of mice with collagen-induced arthritis (104). Therefore, in this complex auto-immune disease, macrophages can both direct and be influenced by the inflammatory environment and cells around them leading to maintaining a pro-inflammatory phenotype and sustaining disease activity.

### Targeting Macrophages in Rheumatoid Arthritis

In RA, preliminary therapeutic intervention is the use of untargeted synthetic disease modifying anti-rheumatic drugs (sDMARDs) such as an immunosuppressing corticosteroid derivatives (e.g. methotrexate) (105). Patients with severe disease who do not respond effectively to this therapy are subsequently given biological disease modifying anti-rheumatic drugs (bDMARDs), which now cover a range of effector molecules in RA such as IL-1, IL-6, IL-17, TNF-α, T cell co-stimulatory molecules, B cell activation and JAK/STAT signaling (106). The use of anti-IL6R or anti-TNF antibodies are the most widely used and have been demonstrated to reduce the level of pro-inflammatory factors in the synovial fluid such as IL-8, IL-1 and MCP-1 (107). However, despite these improvements in the disease, approximately 40% of patients treated with bDMARD therapy show little or no efficacy (108), and as such other novel therapeutic approaches are being considered. Disappointingly, specific inhibitors of pro-inflammatory signaling pathways which are known to drive the secretion of these factors in macrophages, such as the p38 MAPK pathway have proved to have little therapeutic effect in clinical trials (109).

As macrophages are one of the primary sources of many of these pro-inflammatory factors (110), targeting macrophage numbers or activity has been proposed to be a prime target to reduce inflammation in this disease (111, 112). Ablating strategies to reduce macrophage numbers in the joint has met with variable success in RA as depletion has been linked to immunosuppression, infection and impaired wound healing (113). Several strategies such as clodronate liposomes (114), photosensitizer-linked nanoparticles (115) or targeting folate receptors (116) have been attempted, but as yet have not been translated to clinical trials. However, these target both anti-inflammatory SMacs and pro-inflammatory MDMs indiscriminately. Therefore, future macrophage therapies will benefit from a greater understanding of the distinct populations within the synovium, their function and potentially identifying markers that could be used to deplete or enhance the activity of individual subsets.

One method would be to reprogram macrophages *in situ* to switch their functional phenotype. We now have a greater understanding of how metabolism in macrophages influences their pro or anti-inflammatory properties, as well as how the RA environment directs this programming (117). RA patients have an increased M1 macrophage profile compared to other arthritides (118) and inflammatory models demonstrate a complex relationship between M1 and M2 subsets depending on the stage of the disease and tissues studied (119, 120). In the inflamed synovium, the predominant macrophage population is characterized by M1-like inflammatory cells which in RA synovium have higher levels of MMPs, pro-inflammatory factors such as TNFα and have a reduced expression of the M2 macrophage associated marker CD209 (121). This imbalance in macrophage subsets is also apparent in the synovial fluid of patients with active disease where there is a significant increase in pro-inflammatory M1 cell numbers (122). M1 macrophages generally have high glycolytic activity whereas M2 type cells have increased oxidative phosphorylation (123). The synovial fluid of RA patients also contains higher levels of glycolytic metabolites and hypoxic markers (124), which may promote the skewing of macrophage physiology in disease. Modulating the metabolic environment of the joint during disease is being studied to assess the potential for therapy. For example, inhibiting glycolysis in models of inflammatory arthritis has been demonstrated to reduce the severity of disease (125). A number of therapeutic strategies are available to modulate the reprogramming of pro-inflammatory M1 to an anti-inflammatory M2 phenotype through targeting metabolic processes in various inflammatory diseases (126). However, directly targeting macrophage metabolism will be challenging in RA as other cell types including synovial fibroblasts have been demonstrated to secrete factors which modulate SMAc metabolism (127). Interestingly, treatment with anti-TNF bDMARDs reduces both hypoxia and glycolysis in the synovium (51).

Alternatively, blocking the influx of monocytes into the inflamed joint has been proposed to restrict the local expansion of the macrophage population (48). However, this form of therapy would have to be localized to the joints affected to prevent the loss of macrophage populations and subsequent side effects in other tissues of the body. New specific effector molecules could be targeted for a macrophage specific therapy including IL-34 (promotes monocyte differentiation to macrophages) or IL-35 (stimulates M2 polarization) (128, 129). An alternative approach to modulating synovial macrophage behavior has been the use of extracellular vesicles from mesenchymal stem cells to induce an anti-inflammatory switch in macrophage polarization in inflamed joints (130, 131).

## The Role of Macrophages in the Induction and Maintenance of Inflammation: Allergic Asthma

### The Immune Response in Allergic Asthma

Asthma is a common and potentially life-threatening disease that imposes a significant burden on patients, their families, the greater community, and health care systems. It is estimated that asthma currently affects about 339 million people worldwide, with a prevalence of 1-18%, depending on the country (132). Although the prevalence of asthma has dramatically increased over the past few decades, there have also been improvements in patient outcomes and reductions in hospitalizations for asthma attacks due to advances in the pharmacological management of this disease.

Allergic asthma is a chronic pulmonary disease characterized by reversible airway obstruction, leading to limited airflow and the manifestation of physiological symptoms, including wheeze and cough. These symptoms are caused by changes to the structure of the large airways (the bronchi and bronchioles), known as airway remodeling, with excess production of mucus and extracellular matrix (ECM), along with hypertrophy, hyperplasia, and hypersensitization of airway smooth muscle (133). These structural changes are driven by persistent inflammation around the airways in response to allergen inhalation; the most common allergens affecting the asthmatic population are derived from pollens, pet dander, cockroaches, and house dust mites (132). The primary factor underlying the development of allergic asthma is an exaggerated hypersensitivity response to allergen (134). Sensitization to allergens in genetically susceptible people results in an IgE-mediated immune response; following exposure, allergen crosslinking of IgE on the surface of mast cells results in the release of mast cell granule contents (histamine, leukotrienes, prostaglandins, and myriad other inflammatory mediators (135), which triggers the infiltration of T helper cells, eosinophils, and macrophages to the site of allergen exposure (134). Additionally, some allergens directly cause damage to the airway epithelium [e.g. Der p1, derived from mites (136, 137)], thereby inducing a danger signal to initiate an adaptive immune response. The establishment of a Th2-polarized inflammatory environment around the airways is characterized by high levels of cytokines (IL-4, IL-5, IL-13), chemokines (CCL5, CCL11, CXCL2, CXCL12), and growth factors (TGF-β, bFGF, VEGF, PDGF-B) (137–139). These soluble mediators, in addition to coordinating and sustaining the immune response to allergen, have profound effects on the structural cells of the airway and directly contribute to the excess mucus production, fibrosis, and airway smooth muscle changes that are directly responsible for the manifestation of asthma symptoms (133).

### Lung Macrophage Diversity and Immune Regulation in Allergic Asthma

Macrophages play critical roles in maintaining the immune response in respiratory inflammation. Macrophages are abundant in the lung, comprising about 70% of the immune cell population (4). Lung macrophages are a heterogenous population of cells, as they may either develop from the differentiation of lung-infiltrating bone marrow-derived monocytes or from the proliferation of resident macrophages of fetal origin (140, 141). Similar to other organs, heterogeneity exists in terms of lung macrophage activation and polarization status, with various populations of macrophages displaying either pro-inflammatory or pro-resolution functions, or a combination of both. As described in other tissues, the extremes of the spectrum of macrophage polarization in the lung have been designated as classically activated (M1) and alternatively activated (M2) phenotypes, reflecting the Th1 and Th2 polarization status of helper T cells (142). The M1 macrophage phenotype is promoted by exposure to the cytokines TNF-α and IFN-γ, as well as pathogen-derived danger signals such as lipopolysaccharide (LPS), leading to the upregulation of mediators associated with the clearance of respiratory pathogens. Conversely, M2 macrophages are induced by IL-4 and IL-13 and are associated with the resolution of inflammation and the clearance of dead cells. However, persistent activation of M2 macrophages in the lung is seen in cases of inflammation induced by helminths such as *Schistosoma mansoni* (143), or by chronic aeroallergen exposure (144). Under these conditions, M2 macrophages have been associated with pathological tissue fibrosis (4, 145). Increased M2 macrophage polarization and activation have been observed in allergic asthma, both in human asthmatics and in mouse models of asthma (146–148), although a better understanding of the molecular mechanisms regulating macrophage polarization is essential to clarify the relationship between allergen exposure, macrophage activity, and the development of airway remodeling and respiratory symptoms in allergic asthma.

The different sources of pulmonary macrophages are reflected in the location and functions of two major lung macrophage populations: alveolar macrophages and interstitial macrophages (4). Alveolar macrophages reside on the epithelial surface of the alveoli and are thus in direct contact with the environment and foreign particles entering the lungs, e.g. bacteria, air pollution particles, and allergens. Alveolar macrophages are for the most part derived from fetal monocytes that colonize the lung shortly after birth, but are replenished by blood-borne monocytes if they are damaged or depleted (141, 149). In contrast, interstitial macrophages reside in the tissue surrounding the airways and have been less well-studied, partially due to difficulties in the identification and isolation of these cells. Recent reports have demonstrated that interstitial macrophages are a diverse cell population than can be divided into three functional subtypes, defined by shared expression of classical macrophage markers, i.e. MerTK, CD64, CD68, and F4/80 as well as phagocytic activity, but differential expression of proinflammatory cytokines, chemokines, MHC Class II, and CD206 (150). The location of these interstitial macrophages throughout the respiratory tree, their ability to polarize toward M1 and M2 phenotypes, and their roles in the development and maintenance of allergic airway disease remain incompletely understood.

Considering that the airway inflammatory microenvironment is rich in Th2 cytokines such as IL-4 and IL-13, which are the primary inducers of M2 macrophage polarization, it is currently thought that M2 macrophages play major pathological roles in allergic asthma. IL-33, which is known to be expressed as a danger signal following allergen-mediated epithelial cell damage (151), can polarize macrophages toward an M2 phenotype (152), in addition to coordinating type 2 helper T cell responses. Other cell types involved in the immune response in allergic asthma, in particular eosinophils, innate lymphoid type 2 cells (ILC2), CD4+CD25+ regulatory (Treg) cells, and mesenchymal stem cells (MSCs) have also been demonstrated to modulate macrophage polarization toward the M2 phenotype (153–156). Certainly, M2 macrophages expressing high levels of CD206 and MHC Class II are abundant in the airways of asthmatics, as these cells have been found to be increased by 2.9-fold in the bronchoalveolar lavage fluid of asthma patients in comparison to the abundance of these cells in healthy control subjects (157). Of the various M2 subsets, M2a macrophages are most relevant to asthma pathology, as these cells are induced by IL-4 and IL-13 and express high levels of CD206 (6). However, it is important to note that the impact of IL-4 on macrophages depends on a number of factors, i.e. the origin of the macrophage (158), the inflammatory microenvironment (159), as well as prior activation (160). Moreover, M2a macrophages express a number of mediators associated with tissue fibrosis, particularly TGF-β (6). Moreover, macrophage-derived cytokines (IL-1β, IL-6, TSLP, IL-33) and chemokines (CCL2, CCL17, CCL22) have been shown to promote Th2 cell differentiation and recruitment (161–164), indicative of a complex form of crosstalk between these immune cell types.

Recent studies on the mechanisms by which human lung macrophages respond to allergen exposure and promote inflammation have revealed that these cells can be directly activated by allergen-derived proteins (165). Gordon et al. demonstrated that house dust mite (HDM)-derived cysteine and serine proteases induce the secretion of apolipoprotein E (APOE) from airway-resident macrophages *via* protease-activated receptor 2 signaling. APOE at these concentrations (≥25 nmol/L) was found to activate the nucleotide-binding oligomerization domain, leucine-rich repeat-containing protein (NLRP) 3 inflammasome and induce IL-1β expression. This study demonstrated that macrophages provide a critical danger signal in response to allergen exposure, namely APOE, and through this mechanism enhance the adaptive inflammatory response to aeroallergens (165). Similar findings were also reported by Tiotiu et al., based on an analysis of sputum macrophages obtained from 104 asthmatics and 16 healthy volunteers in the U-BIOPRED (Unbiased BIOmarkers in PREDiction of respiratory disease outcomes) asthma cohort (166). In this study, the gene signatures for differentially stimulated macrophages, i.e. lung tissue-resident macrophages, as well as classically and alternatively activated macrophages, were assessed by gene set variation analysis. It was found that, although macrophage numbers were significantly lower in severe asthmatics compared to mild/moderate asthmatics and healthy controls, the M2 signature was much higher in severely ill patients. Interestingly, macrophages from severe asthmatics were also enriched for eicosanoid biosynthesis and showed evidence of increased mitochondrial ROS production, implicating macrophages in the maintenance of inflammation and the induction of oxidative stress in cases of severe disease (166). Intriguingly, this study found significant changes in tissue-resident macrophage signature enrichment according to asthma severity. Most of the genes within this cell population signature are involved in mitochondrial function, lipid metabolism, tissue homeostasis, and apoptosis. It has been suggested that the dynamic polarization of lung tissue-resident macrophages during inflammation is highly dependent on mitochondrial activity, i.e. increased levels of oxidative phosphorylation early in the immune response facilitates cytokine expression and cell migration, whereas during the resolution phase, macrophages are considerably less active (166). The data from U-BIOPRED indicate that, in both healthy subjects and mild/moderate asthmatics, tissue-resident macrophages exist in a state of readiness, which is not unexpected given their exposure to the external environment. However, this homeostasis breaks down in severe asthma, with evidence of increased expression of inflammatory mediators, in particular Th2 cytokines such as IL-4 and IL-13 (166). Clearly, gaining a deeper understanding of the functional diversity and plasticity of macrophages in allergic asthma will be important for developing more effective therapeutic strategies based on disease phenotype and allow for a more personalized medicine approach to the treatment of asthma.

### Mechanistic Insights Into Macrophage Function in Allergic Airway Disease Using Mouse Models

As a corollary to these studies on macrophages in human asthmatics, a number of recent, innovative studies have exploited mouse models of asthma to provide mechanistic insight into the modulation of disease pathways by pulmonary macrophages. Branchett et al. (167) performed bulk RNA sequencing on airway macrophages obtained using flow cytometric sorting (CD11c^+^ SiglecF^+^ CD64^+^ CD45^+^ SSC^hi^) to determine that repeated exposure to the aeroallergen HDM induced a macrophage phenotype characterized by increased expression of genes associated with antigen presentation, oxidative metabolism, inflammatory cell recruitment, and tissue repair, and downregulation of genes associated with the cell cycle, cytoskeletal function and antimicrobial immunity (167). Of particular interest was in the increased expression of chemokines by HDM-stimulated macrophages, with elevated expression of chemoattractants for key cell types involved allergic asthma, i.e. Th2 cells (CCL17 and CCL8) and eosinophils (CCL24), indicating a primary role of tissue-resident macrophages associated with the airways in regulating the immune response to allergen (167). The results furthermore infer a role for airway macrophages in stimulating incoming Th2 cells to sustain the inflammatory response, given their increased expression of MHC Class II, likely driven by high levels of IL-4 and IFN-γ under these inflammatory conditions (168). Additional evidence for a direct impact of allergen exposure on macrophages and the resulting alterations in immune mediator expression has recently been provided by Henkel et al. (169). Following either direct stimulation of monocyte-derived macrophages with HDM or respiratory delivery of HDM to mice, macrophages were found to undergo fundamental reprogramming of lipid mediator metabolism, displaying remarkable plasticity in terms of prostanoid (high expression *in vitro*) and eicosanoid (high production *in vivo*) inflammatory mediator production (169). Taken together, it is clear from recent studies that allergen exposure induces a pathogenic phenotype in macrophages, characterized by abundant production of proinflammatory chemokines, cytokines, and lipid mediators. Considering that these mediators are implicated in treatment-resistant allergic asthma, investigating methods to control the activity of pathogenic macrophages may yield a significant clinical benefit in severe asthmatics.

The role of hypoxia in driving critical changes to macrophage phenotype has recently been investigated by Sokulsky et al. (144). Also using an HDM-driven model of allergic airway disease, this study demonstrated the role of glutathione S-transferase omega class 1 (GSTO1-1) in the regulation of macrophage redox state and the LPS/TLR4/NLRP3 signaling pathway. Intriguingly, GSTO1-1-deficient mice exposed to HDM had greater numbers of eosinophils and macrophages and elevated levels of eotaxins in comparison with similarly exposed wild type mice, as well as significantly higher expression of M2-related genes such as Ym1. This elevation in the expression of markers of type 2 immunity were found to be regulated *via* HIF-1α, indicating a previously unrecognized role for the induction of hypoxia in mediating the severity of allergic airway inflammation (144). As a similar role for hypoxia in driving a macrophage phenotype capable of exacerbating glioma progression has recently been revealed (170), it is clear that further studies on the interplay between hypoxia and the induction of a pathological M2 phenotype may yield interesting and novel therapeutic targets.

Some recent studies have attempted to exploit the immune regulatory role of alveolar macrophages to control allergen-induced airway inflammation. In contrast to the pathological nature of M2-polarised tissue-resident macrophages associated with the large airways, alveolar macrophages have been shown to provide a type of immune barrier function, with the capacity to suppress type 2 immune responses and prevent airway hyperresponsiveness in allergic airway disease mediated by respiratory exposure to ovalbumin or house dust mite (171–173). Recently, Li et al. employed an adoptive transfer strategy to modulate inflammation in the lung in response to ovalbumin inhalation (174). In this study, infusion of clodronate-encapsulated liposomes to deplete alveolar macrophages led to an aggravated inflammatory response to allergen exposure. Moreover, adoptively transferred alveolar macrophages acquired an M2-type phenotype and suppressed M1 responses, but were found to play crucial roles in enhancing the inflammatory response to allergen. Crucially, the acquisition of M2 characteristics was found to be mediated *via* the ATP/P2X7r axis, suggesting that pharmacological intervention to modulate purinergic signaling pathway may be clinically beneficial (174).

Other recent studies have attempted to delineate the mechanism of immune suppression by alveolar macrophages. Miki et al. interrogated the downstream effects of apoptotic cell engulfment by alveolar macrophages in the context of HDM-driven allergic airway inflammation and found that enhancing the phagocytic capacity of alveolar macrophages led to increased expression of immunosuppressive mediators and the induction of regulatory T cells in the lung (175). Intratracheal infusion of apoptotic thymocytes in the context of HDM exposure had the effect of suppressing Th2 cytokine expression and reducing airway eosinophilia, thought to be mediated by induction of the suppressor of cytokine signaling molecule SOCS3 and adenosine receptors in alveolar macrophages (175). The administration of apoptotic macrophages has also been investigated as a means of inhibiting bleomycin-induced lung inflammation and fibrosis, with the intriguing finding that the additional delivery of simvastatin along with apoptotic macrophages further enhanced efferocytosis in alveolar macrophages and moreover increased PPARγ activity, induced hepatocyte growth factor and interleukin-10 expression, and decreased the expression of factors associated with epithelial to mesenchymal transition (176). Additionally, a study by Woo et al. recently revealed a critical role for GM-CSF signaling in alveolar macrophages in the regulation of allergic inflammation (177). Using an alveolar macrophage-specific conditional knockout strategy and HDM exposure in mice, these authors demonstrated that disrupted GM-CSF signaling in alveolar macrophages led to reduced airway inflammation and lower expression of type 2 cytokines following allergen exposure. Mechanistically, these genetic alterations to GM-CSF signaling in alveolar macrophages were found to induce metabolic reprogramming and a loss of mitochondrial homeostasis *via* PPAR-γ, resulting in deficient TNF-α and MHC Class II expression and reduced antigen uptake following allergen exposure (177). Taken together, these studies demonstrate that the manipulation of alveolar macrophages in allergic airway disease may be an attractive therapeutic target.

### Pharmacological Strategies Targeting Macrophages in Allergic Asthma

A number of recent studies have evaluated the utility of currently available therapies for modulating macrophage function in allergic asthma. Maneechotesuwan et al. have conducted a series of randomised, double-blind, placebo-controlled studies in moderate/severe asthmatic patients treated with an inhaled corticosteroid alone or in combination with oral statin therapy to investigate the mechanism by which this drug combination exerts a remarkable anti-inflammatory effect in allergic asthma, resulting in robust IL-10 expression and the induction of regulatory T cells (178, 179). In their most recent clinical trial and supporting *in vitro* studies, Maneechotesuwan et al. determined that corticosteroids and statins synergistically suppress autophagy in airway macrophages *via* inhibition of the PI3 K-Akt/mTOR signaling pathway, resulting in greatly enhanced IL-10 production by macrophages and improved asthma control (179). The consequences of this elevation in IL-10 expression, especially regarding its effects on eosinophil survival and the production of type 2 proinflammatory cytokines (IL-4, IL-5, IL-13) certainly warrant further investigation.

Non-antibiotic macrolides have been under investigation for some time as an alternative treatment modality for steroid-resistant asthmatics, particularly during exacerbations (180), although the mechanism of action of this intervention has not been fully elucidated. Using the HDM exposure model of allergic airway disease in mice, Sadamatsu et al. (181) recently showed that the non-antibiotic macrolide EM900 reduced airway inflammatory cell numbers and airway hyperreactivity when delivered therapeutically. Mechanistically, EM900 was found to significantly decrease the expression of asthma-associated inflammatory mediators such as IL-5, IL-13, CCL5 and CXCL2 by lung interstitial macrophages, *via* the suppression of NF-κB and p38 signaling (181). Additional pre-clinical and clinical studies on EM900 and other drugs of this class are needed to clarify the role of non-antibiotic macrolides in the treatment of severe asthma.

## Macrophages and the Resolution of Sterile Inflammation

Macrophages are important cells in the context of inflammation, whether this be resident macrophages that may act to promote inflammation in response to tissue challenge or recruited macrophages that may be also important in the promotion of repair responses. It is the dynamic and plastic nature of these important myeloid cells that puts them at the heart of the inflammatory process and, consequently, it is the control of these cells that offers great opportunity for novel potential interventions within inflammatory disease.

Whilst much is known about the signaling pathways that can lead to the classic pro-inflammatory macrophage phenotype, an important feature within a defense response, the switch of these macrophages to alternatively-activated macrophages remains relatively poorly understood (182). However, this switch is an essential control point if inflammation is to achieve its desired outcome of returning a challenged tissue to its pre-inflamed, fully functional state. A failure in this control point leads to chronic inflammation and ultimately tissue scarring and loss of tissue function.

Within experimental models of inflammation, the kinetics of the cellular inflammatory process are highly predictable and coordinated, and in the case of granulocytic inflammatory responses, cell death of inflammatory cells has an important place within the control of macrophage phenotype and the resolution of inflammation.

### Efferocytosis: The Finding, Binding, and Grinding of Cell Corpses

As inflammatory cells die by apoptosis, the dying cells communicate their presence to surrounding cells and promote the recruitment of macrophages to the sites of cell death where they are then able to remove cell corpses in a process known as efferocytosis (183). The mechanisms by which apoptotic cells communicate their presence through the release of apoptotic cell-derived factors is a relatively new area of study.

The blebbing of the plasma membrane (zeiosis) and release of apoptotic cell fragments in the form of extracellular vesicles (EV) is a defining feature of apoptosis (184). Recently these EV have been defined as “find me” factors that recruit macrophages to dying cells *via* ICAM-3 and the chemokine CX3CL1 (185–187). EV are complex multi-molecular compartments that share features with their parent cell and can modify immune responses (188, 189). Furthermore, all EV have the potential to be “active EV” through the carriage of enzymes; this can have profound effects on the functional significance of EV through modification of the extracellular matrix (190–192) and through limitation of tissue damage (193). As EV can be of dramatically varying sizes, consequently there is the potential for them to be significant independent metabolic compartments (194); this is an area that requires additional research, particularly for the analysis of inflammation controlling enzymes (195). Soluble “find me” factors are also released from dying cells and a range of apoptotic cell-derived molecular mediators of macrophage recruitment have been identified including nucleotides (ATP, UTP) (196), lipids (sphingosine-1-phosphate, lysophosphatidylcholine) (197, 198), and chemokines (CX3CL1) (187).

On arrival at a site of cell death, macrophages bind apoptotic cells through receptor-ligand interactions; these have been extensively reviewed elsewhere (199–201). The prime apoptotic cell “eat me” ligand is the exposed phospholipid phosphatidylserine (202, 203), which can be bound directly by phosphatidylserine (PS) receptors (e.g. BAI1, TIM-4, stabilin-2) (204–206) or indirectly through the use of soluble bridging molecules Gas-6, protein S, and MFG-E8) (207–209), which in turn bind to macrophage integrins and TAM receptors (210–212). However, there are other, relatively ill-defined, changes that function as clearance ligands (i.e. “eat me” signals) including changes in glycosylation, exposure of calreticulin (213), and functional changes in ICAM-3 (214). Notably, these “eat me” signals are opposed by “don’t eat me” signals that provide balance to this important process through inhibitory responses (215). CD47 is a good example of such a negative signal that ligates SIRP-α on macrophages. When this receptor-ligand interaction is inhibited, efferocytosis is promoted (213, 216).

Many receptors for apoptotic cells have also been identified (217). Some appear to function in individual stages of efferocytosis (e.g. CD14 and binding) (218–220), while others appear functionally redundant; this likely explains why not all phagocytes carry the same panel of apoptotic cell uptake molecules. Such diversity in apoptotic cell receptor carriage leads to an extra dimension within the consideration of phagocyte plasticity. Perhaps those receptors with the greatest significance are those that promote the next ingestion stage in the clearance process, i.e. phagocytosis as a result of cytoskeletal organization. These include PS receptors: BAI1 (204), the TAM (Tyro3, Axl, Mer) family of receptors (221), TIMs (222), and integrins (223).

Following phagocytosis, the cell corpse can be digested and the phagocyte can respond with the production of anti-inflammatory mediators, which play an important role in the resolution of inflammation.

### Efferocytosis, the Avoidance of Disease, and Modulation of Macrophage Phenotype

There is consensus that apoptotic cell clearance is a beneficial process which, through its efficiency, avoids secondary necrosis and unwanted inflammatory and autoimmune consequences. The brief summary above belies the very significant amounts of research that have sought to define the mechanisms by which apoptotic cells and macrophages (and a wide range of other professional and non-professional phagocytic cells) interact to effect corpse removal and crucially realize the functional significance of this process – immunomodulation. In achieving this, efferocytosis results in a significant phenotypic shift within macrophages towards an alternatively activated/anti-inflammatory phenotype (182). These macrophages are then active in promoting the necessary repair responses that drive tissue homoeostasis.

During efferocytosis, there is activation of a molecular switch that drives the fine balance in inflammatory mediators away from a pro-inflammatory response and towards a pro-repair response; lipid mediators of inflammation (e.g. prostaglandin E2 and D2) are crucial in this switch (224–226). The benefit of this switch towards resolution in the context of “classic” sites of inflammation is clear, though it is noteworthy that the same processes can drive pathology in other contexts where inflammation is a driving force, e.g. cancer. For example, the alternatively-activated M2 macrophage can help to promote tumourigenesis (227); this helps to highlight the opposing faces of an inflammatory response.

A failure in efferocytosis leads to disease with chronic inflammatory conditions. Within the lung, this contributes to asthma (228, 229), leading to exacerbated disease (230, 231). This has been attributed at least in part to a reduction in galectin 3 which is involved in the control of efferocytosis (232). Recognition of apoptotic eosinophils through MerTK has been shown to be an important efferocytosis pathway to promote the resolution of allergic airway inflammation (233). Failed efferocytosis also contributes to chronic obstructive and idiopathic pulmonary disease, cystic fibrosis, and airway inflammation (231). Similar consequences are seen with any failure of efferocytosis in most if not all tissues, with profound disease consequences, including tissue and systemic autoimmune conditions (234).

A number of mechanisms have been proposed for the way in which apoptotic cell clearance by macrophages prevents inflammation. An important process relies upon the production of the anti-inflammatory cytokines IL-10 and TGF-β1 and inhibition of pro-inflammatory cytokines (235, 236). Efferocytosis induces IL-10 production in macrophages (237), which in turn helps to support macrophages to clear apoptotic cells efficiently (238). This positive control system drives improved apoptotic cell clearance through the activation of LXR, a nuclear receptor that promotes TAM receptor activity (239), and promotes an effective M2 macrophage phenotype to support resolution of inflammation. More recently, the type I interferon IFN-β has been shown to be induced by both TGF-β1 and apoptotic cells, becoming intricately associated with the resolution of acute inflammation through the control of neutrophil apoptosis and efferocytosis, rather than neutrophil recruitment (240). This work further highlights the complexity of macrophage phenotype at sites of inflammation, noting the concept of “satiety” in macrophages that had gorged on apoptotic cells prior to becoming an established non-phagocytic resolution phase macrophage producing high levels of IFN-β which is involved in IL-10 expression. The full impact of this IFN-β “circuit” requires further study. Recently, the cell corpse “meal” has also been shown to provide the necessary fuel for fatty acid oxidation to support macrophage polarization for tissue repair (241).

Efferocytosis also drives an important switch in the production of small lipid mediators of inflammation, termed eicosanoids. They comprise a wide range of mediators, including the prostanoids (e.g. prostaglandins, thromboxanes), leukotrienes, resolvins, and lipoxins. These mediators are the focus of much research and are a family of signaling molecules generated from the oxidation of polyunsaturated fatty acids. They are a growing family of molecules that range in their function from pro-inflammatory to anti-inflammatory, such that the balance of these mediators can be crucial in promoting resolution. The cardinal signs of inflammation (redness, heat, swelling, and pain) are driven by these agents, and prostaglandin E2 is particularly important. During efferocytosis, a particular subclass of these eicosanoids is produced, known as specialized pro-resolving mediators (SPM), including members of the resolvin D and E series (produced from the omega-3 fatty acids DHA and EPA, respectively) (242). The production of SPM resolvins and protectins occurs in line with a switch away from the production of the pro-inflammatory prostaglandins and leukotrienes (226), consequently shifting the fine inflammatory balance towards resolution with M2 macrophages exhibiting higher SPM levels (e.g. lipoxin A4) and lower inflammatory eicosanoid levels.

There is much still to learn about the impact of cell death in the control of innate immune responses. Whilst the use of experimental models of inflammation are powerful systems for the dissection of inflammatory signaling pathways, it is of great value to also examine disease settings. A number of conditions are associated with non-resolving inflammatory responses. For example, atherosclerosis where efferocytosis is reduced due to loss of macrophage TAM receptors (243–245), and this is further associated by reduced SPM production collectively resulting in failed resolution, larger lesions with necrotic cores (246). Cancer also represents a powerful pathological opportunity for study of inflammation, cell death and macrophage phenotype. Whilst it is beyond the scope of this piece to review fully the breadth and depth of work regarding tumor-associated macrophages, there are some important points worthy of consideration. The phenotype of macrophages associated with tumors can dramatically change the outcome of the lesion, with inflammatory, M1-like macrophages considered as tumor suppressive and pro-repair, M2-like macrophages being supportive of tumor growth. Of relevance here is the observation that tumors, paradoxically, can have high levels of cell death and this can be associated with particularly aggressive tumors (247). This point of cross talk between these dying cells and tumor associated macrophages, both directly and *via* extracellular vesicles, can promote a “pro-repair” phenotype in macrophages (i.e. M2-like or alternatively-activated) (248) and the contribution of these macrophages to support of the tumor has been proposed as an onco-regenerative niche (227, 249).

### Extracellular Vesicles and the Control of Innate Immune Responses

The molecular mechanisms upstream of corpse removal (the “find me” phase) remain relatively poorly defined. Powerful communicating agents known as extracellular vesicles (EV) have become the focus of much research over recent years, and their ability to modulate the innate immune response and repair of tissues has attracted much attention in recent times. These EV are derived from all cells and can be produced from a variety of cellular locations. They are membrane-enclosed compartments that are released from viable cells and apoptotic cells and can be derived from intracellular sources (i.e. multivesicular body) or the plasma membrane by an increasing range of identified processes [reviewed by (188)]. The impact of these EV on immune responses has been the subject of much research, highlighting roles in antigen presentation, inflammatory responses, and infection processes (189, 250–252). EV from apoptotic cells have also been proposed to have profound immunomodulatory effects.

EV have been given many names in research across different disciplines, but within the field of cell death, they have been termed “apoptotic bodies” and more recently apoptotic cell-derived EV (ACdEV or ApoEV) to capture the probable diversity in these EV and their routes of generation. Since the seminal work in 1972 (184) where apoptotic cells and EV were noted to be of varying size, to more recently within the field of EV, the term apoptotic bodies has been used to describe only the largest of the vesicles (>1 µm) released from dying cells. Within the increasing number of studies of apoptotic cell-derived extracellular vesicles, it is clear that these are complex, highly functional, immune-modulating mediators.

A key part of resolving inflammation is to prevent new inflammatory cell recruitment (e.g. to exhibit “keep out” signals to exclude granulocytes); this has been shown to occur in a number of ways. First, through apoptotic cells releasing lactoferrin to act as a “keep out” signal (253) and also as a mechanism to inhibit pro-inflammatory signaling (254). Indeed, lactoferrin-derived fragments and peptides generated following the uptake of apoptotic neutrophils by macrophages are a critical immunomodulating component (255). Additionally, by the sequestration of chemokines to destroy chemoattractive gradients (256), but also through the release of anti-inflammatory factors (e.g. cytokines and SPM), with TGF-β1 capable of inducing anti-inflammatory SPM whilst reducing pro-inflammatory lipid mediators (257). These SPM are also capable of preventing further inflammatory cell infiltration (258) whilst stimulating recruitment of monocytes to promote efferocytosis and resolution (259).

Apoptotic cell-derived EVs act to recruit phagocytes to sites of cell death (185) *via* mechanisms that have been shown to include intercellular adhesion molecule 3 (186) and the chemokine CX3CL1 (187). The role of EV in SPM carriage and function is becoming clear and is the focus of much research. EV have been shown to shuttle these eicosanoids (260–263), and all EV will be sources of the necessary esterified fatty acid substrates for eicosanoid synthesis (264). The importance of this, especially within apoptotic cell-derived EV, remains the focus of ongoing research.

EV are complex macromolecular structures, and the full functional significance of their composition remains to be elucidated. The exposure of phosphatidylserine at the surface of these vesicles is similar to that seen on apoptotic cells, where this phospholipid is an important immune modulating factor driving macrophage phenotypic switch (202, 236, 265, 266). It is therefore likely that EV are similarly capable of changing macrophage phenotype *via* this mechanism, though there is still a gap in our knowledge in relation to the precise molecular mechanisms and the extent to which EV can affect the switch alone, without the contribution of the apoptotic cell corpse. A recent study has suggested that the induction of TGF-β1 occurs in responses to ACdEV and drives macrophages towards an anti-inflammatory phenotype in experimental colitis (267). Furthermore, the known release of IL-10 by macrophages in response to apoptotic cells may also drive EV release from other cells (e.g. dendritic cells) that are also capable of inhibiting inflammatory responses (268). This further highlights the complexity in the system where EV effects may be direct or indirect in the control of responses. EV from apoptotic cells have been shown to induce apoptosis in macrophages, thereby amplifying the ACdEV response and potentially suppressing inflammatory responses (269). Again, the study of pathology may shed further light on the precise molecular mechanisms by which EV may exert their effects on macrophages and the consideration of cancer as a non-resolving inflammatory lesion will be of value. EV derived from tumor cells, including endogenous apoptotic cells, have been proposed as key regulators in tumor progression (227) and detailed study of the composition-function relationships of these EV with modulation of inflammatory responses would be of value. For example, the SPM microenvironment (either free or in EV) may be important in supporting a beneficially-suppressed inflammatory environment that promotes anti-tumor activity [recently extensively reviewed in (270)].

Extracellular vesicles carry a tantalizing array of different molecular species, including proteins, lipids, and nucleic acids. Interestingly, it is now established that EV can also carry enzymes (190–193, 195); this has led to the possibility of active EV that may change throughout their life cycle. The presence of immune-modifying enzymes within EV is the focus of our current research. It is however established that pro-fibrotic enzymes such as transglutaminase 2 are released during inflammatory responses and these drive a pro-fibrotic disease process (271). The balance between repair responses and fibrotic responses and the ability of extracellular vesicles to change this balance remains a gap in our current knowledge. However, this is precisely the point at which novel therapies are focused so as to control macrophage phenotype for therapeutic gain.

It is notable that EV have been shown to promote efferocytosis, thus supporting the resolution of inflammation. Crucially, they also drive a change in the lipid mediator profile of macrophages (272) by modulation of the expression of the required enzymes for SPM production (273). It seems likely that EV act as an appetizer for macrophages ahead of their main “meal” of a cell corpse, and that they may be a key functional mediator in promoting the necessary macrophage phenotypic shift required to effect strong pro-resolution responses. EV also represent a mechanism by which macrophages can be supplied with SPM precursors and pre-formed mediators that may further support their immune-modulating role (242). It remains unclear whether apoptotic cell-derived EV, like apoptotic corpses themselves, drive robust inflammation-controlling responses or whether they must act in concert with cell corpses to achieve the full anti-inflammatory/pro-repair responses required for the resolution of inflammation.

Whilst dying cells and their vesicles are capable of interacting with the innate immune system to support pro-resolution responses, EV from a range of other cells including mesenchymal stem cells also appear to function in a similar manner. MSC-derived extracellular vesicles are the focus of much attention within the field of regenerative medicine, and it is notable that the responses that they seek to induce within local tissues and through modulation of macrophages are those same responses that occur naturally in response to apoptosis, responses that promote the resolution of inflammation and repair responses. This raises the possibility that extracellular vesicles from viable and dying cells function in a similar manner, but that it is perhaps the larger dose of extracellular vesicles produced during apoptosis that exerts the strongest inducer of a macrophage phenotypic switch. In this context, it is likely that apoptotic cell-derived extracellular vesicles have two functions: one of macrophage recruitment and a second to induce phenotypic change towards a pro-repair phenotype, the latter supported by cell corpse clearance.

### Efferocytosis and the Control of Adaptive Immune Responses

Macrophages also play critical roles in the resolution of adaptive immune responses, predominantly *via* the uptake of apoptotic leukocytes, neutrophils and eosinophils. However, despite these cells presumably acquiring an M2 phenotype under these conditions, it is difficult to explain this “pro-resolution” activity following efferocytosis in asthma, as M2 macrophages have been intimately tied to the induction of tissue fibrosis. While recent studies have found that encounters between apoptotic leukocytes and macrophages contribute to the clearance of cell debris, while at the same time inducing immune silencing in macrophages, some aspects of M2 cell activity may further contribute to disease pathology by exacerbating the inflammatory response and generating factors that contribute to tissue fibrosis (274, 275). Interestingly, a direct comparison of macrophage subsets during the resolution of inflammation has shown that satiated macrophages downregulate genes associated with fibrosis, while non-phagocytic macrophages are associated with processes such as migration, oxidative phosphorylation, and mitochondrial fission. Notably, the conversion to a satiated state has been found to induce a reduction in the expression of extracellular matrix constituents associated with tissue fibrosis (276). Thus, macrophage satiation during the resolution of inflammation seems to bring about a transcriptomic transition that resists tissue fibrosis and oxidative damage while promoting the restoration of tissue homeostasis to complete the resolution of inflammation. Taken together, M2 macrophages appear to be paradoxically involved in both the induction of fibrosis and its resolution, suggesting the need for further research in this area to clarify these mechanisms leading to an anti-fibrotic state of macrophage satiety. Certainly, the resolution of inflammation *via* efferocytosis, particularly relevant to the clearance of neutrophils (277) and eosinophils (278), is a vibrant field of research that has been thoroughly reviewed elsewhere (279).

## Conclusions

It is evident that macrophages are essential for both homeostasis and disease pathology. In this review, we have focused on the origins, differentiation, and functions of tissue macrophages, with a particular focus on the role of macrophages in chronic sterile inflammatory diseases of the joint and lung. Additionally, we have emphasized the crucial role of macrophages in the control and resolution of tissue inflammation. Although many questions remain unanswered regarding the precise molecular and cellular mechanisms involved in macrophage-mediated pathology, we have highlighted recent efforts to target macrophage activity using small molecules, biologics, and extracellular vesicles. With the increasing resolution of phenotyping techniques, a deeper understanding of macrophage subsets and their plasticity over time and space will certainly contribute to the development of effective macrophage-targeting therapies, with an expected improvement human health.

## Author Contributions

All authors contributed to the article and approved the submitted version.

## Funding

The authors gratefully acknowledge financial support from the UK Biotechnology and Biological Sciences Research Council (BB/S00324X/1).

## Conflict of Interest

The authors declare that the research was conducted in the absence of any commercial or financial relationships that could be construed as a potential conflict of interest.

## Publisher’s Note

All claims expressed in this article are solely those of the authors and do not necessarily represent those of their affiliated organizations, or those of the publisher, the editors and the reviewers. Any product that may be evaluated in this article, or claim that may be made by its manufacturer, is not guaranteed or endorsed by the publisher.
